# Slip in adhesion tests of a Kaolin clay

**DOI:** 10.1140/epje/s10189-021-00107-9

**Published:** 2021-08-11

**Authors:** M. J. Hayes, M. I. Smith

**Affiliations:** School of Physics, University of Nottingham, University Park, Nottingham, NG7 2RD UK

## Abstract

**Abstract:**

Adhesion tests were performed on concentrated suspensions of Kaolin clay. At low concentrations samples formed conical deposits on both the top and bottom plates with the central region narrowing to a filament before undergoing breakup. In contrast high concentration samples deformed as a cylinder before apparently fracturing into two pieces. As the concentration of the samples was increased the samples underwent quite different forms of slip which it is shown can be deduced from their respective force distance curves. The type of slip behaviour for a given concentration of clay could be modified with changes to surface roughness, the initial compressive load prior to an experiment and with the separation velocity of the plates. The different slip characteristics appear to arise from the concentration dependent way in which particles interact with the rough surface topography.

**Graphic Abstract:**

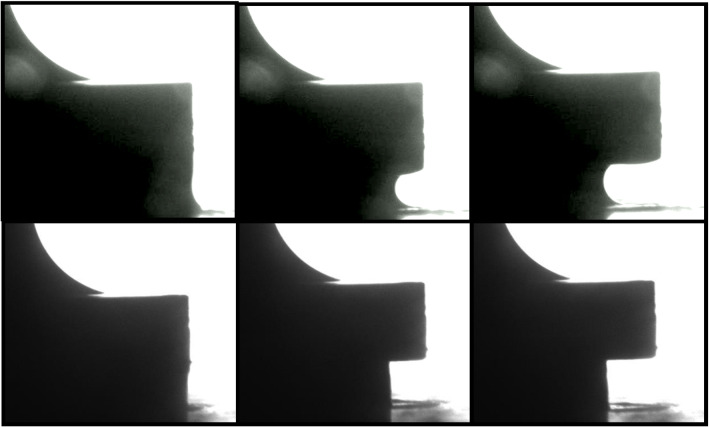

**Supplementary Information:**

The online version supplementary material available at 10.1140/epje/s10189-021-00107-9.

## Introduction

Everyday fluids often give rise to complex flow behaviours that do not fit into the regular categories of liquid and solid. When some fluids are subjected to a stress, below a critical yield stress ($\sigma _{{Y}}$), they may exhibit solid-like behaviour, whilst above this threshold the samples flow or deform [1, 2]. These yield stress fluids include a vast array of commonly used materials such as gels, foams and pastes which find industrial applications in the manufacturing of food, cosmetics and construction [3, 4]. Examples are also common in biology (e.g. snail slime [5], protein gels [6]) and some geological phenomena such as yielding of quicksand [7] and lava [8].

Whilst many studies have focussed on simple shear measurements of yield stress fluids using standard rheometrical techniques, there are many alternative flows frequently used in practical applications. A number of studies have focussed on model systems that approximate these more applied flows such as a flow of liquid down an inclined slope [9], motion of an object through a liquid, [10] coating [11] and capillary flows [12], a rod being drawn from a container [13], squeeze flows [14] and adhesion tests [15]. Interpreting the significance of different features of these flows and understanding how these tests relate to more standard rheological measurements remains a challenge, particularly for yield stress fluids.

In adhesion testing, the focus of this study, a thin layer (${{h}}_{0}$ thick) of fluid is confined between two circular plates of radius ${{R}}_{0}$ such that the initial aspect ratio, ${{h}}_{0}/{{R}}_{0}$, is small. The normal force on the upper plate is then measured as it is slowly moved upwards. A number of studies have focussed on this type of flow which is used extensively in quality control applications of food stuffs [16] but is also quite similar to the geometry encountered when a flat tool is withdrawn from contact with a fluid, e.g. knife and butter, trowel and mortar [17]. There has been significant research on two main aspects of this problem. Firstly, there has been interest in the deposited patterns left behind on either plate. These patterns can be conical deposits, rough multi-peaked fracture surfaces [18] or viscous-fingering induced tree-like structures [19–22]. Secondly, a number of studies have focussed on understanding how the measured force (*F*) scales with plate separation (*h*) [15, 19, 23]. This allows one to calculate the total work of adhesion. The measured scaling is known to depend strongly on the traction conditions experienced by the fluid at each plate. For a yield stress fluid deformed quasi-statically, resulting in solid-like behaviour, if the surfaces are very rough such that the fluid is pinned $F\propto h^{{-}2.5}$, whilst under conditions corresponding to perfect slip $F\propto h^{{-}1}$ [23]. In this study, we also examine the role of slip in the measured forces. However, our focus is on the transient behaviour during the early stages of the adhesion measurement. This stage is important since it determines the separation of the plates at which the maximum force is measured.

Measurements made during the transient start-up of a rheological measurement can exhibit differences from steady state flows. When a yield stress fluid is first subjected to strains below some critical value $\gamma _{{c}}$ ($\sim 0.1{-}0.3$) it typically exhibits a visco-elastic response, whilst above this critical deformation the samples yield, displaying visco-plastic behaviour [10]. In systems where the particles have attractive interactions this can be followed by a second far less obvious yield point at higher strains [24]. Under some conditions the initial stress–strain curve during the transient start-up may exhibit a stress overshoot before settling down to a steady-state [25]. The existence of this stress overshoot in some systems has also been strongly linked to the development of wall-slip or transient shear banding which affect the apparent measured properties of yield stress fluids [26].

Slip results in a local velocity that varies over some small but mesoscopic distance from the boundaries. In the case of yield stress fluids composed of soft deformable particles slip occurs via the creation of elastohydrodynamic lubrication layers [2, 27]. In contrast slip in yield stress fluids of concentrated rigid non-Brownian particles arises from a local depletion of particles, lowering the concentration of the sample near to the wall [2, 19, 27]. However, the nature of slip can vary depending on the boundaries and imposed shear rate [28, 29]. Under certain conditions the apparent slip is actually a result of shear banding, with the shear localised to a thin layer near to the boundaries [27]. At the microscopic level slip is controlled by the complex interactions of the particles with the rough topography and surface chemistry [30]. Whilst it has been possible to correlate changes in slip with suspension microstructure for certain select cases [31], understanding this complex relationship is still a challenge for most systems.

Traditionally rheological measurements have assiduously attempted to avoid or suppress slip of the fluid at boundaries with the use of roughened surfaces. However, in many real-world applications, slip can be beneficial, reducing the stress required to move a sample relative to a boundary [27]. Particularly for the case of high solid dispersions, slip at interfaces controls the sample flow properties and can prove unavoidable [27, 32]. An understanding of the factors governing the type of slip in an adhesion measurement or its effect on the measured force–distance curves, would be useful for practical applications.

A number of studies have performed adhesion tests on clay [22] or high solid content pastes [17, 18, 33]. Different break-up regimes were reported, resulting in fingering instabilities or void growth mediated fracture. However, these studies reported that the samples underwent no or very little slip. In this work, we perform adhesion tests using kaolin clay where slip is possible. Focussing on the initial stages of the flow we find that changes in the observed slip of the fluid at the boundaries result in qualitatively different force–distance curves. We show how the nature of the slip during the initial stages varies with clay concentration, boundary roughness, the initial compressive load and deformation rate. We also discuss to what extent these initial differences play a role in the break-up behaviour of the samples.

## Materials and methods

### Sample preparation

Kaolin clay suspensions (RM1018 Grolleg, Scarva) with solid contents ranging between 45 and 65 wt% were prepared with deionised water. The volume fraction of each clay ($\phi \sim 0.31{-}0.47$) was estimated by measuring the mass loss of water from known volumes of sample heated in a vacuum oven at $120^{\circ }\hbox {C}$ until constant mass was obtained. SEM images showed that at these concentrations the clay platelets are tightly packed in layered structures (see supplementary material). The clay was comprised of agglomerates with a highly heterogeneous range of sizes ($\sim 200\hbox {nm} {-}20\,\upmu \hbox {m}$). Stock batches were stored under refrigeration in large, sealed containers at $5^{\circ }\hbox {C}$ for a maximum of one month. Prior to measurement, 50 ml of sample was carefully removed and vortex mixed overnight to ensure uniformity. Measurements were also performed using a 2 wt%, 10 kDa Carbopol suspension Ultrez 10, neutralised by dropwise addition of 0.1M NaOH and dispersed using a magnetic stirrer at a spin speed of 200 rpm.

### Rheological measurements

All measurements were made with a Kinexus Pro rheometer (Malvern Panalytical) equipped with a 20-mm diameter parallel plate upper geometry and a 10-mm thick Perspex block was used as the lower plate. The surface of the top plate was covered with a 20 mm diameter Perspex disc which had been roughened by sandblasting with Saftigrit Brown 100 (Guyson, $\sim 125 \, \upmu \hbox {m}$). The roughness was chosen so as to be comparable to the largest particle size in the clay (in monodisperse particle pastes this is believed to provide the optimum conditions to reduce slip [27]). The Perspex block was also sandblasted to match the surface characteristics of the upper plate. The samples were filmed (Point Grey, USB Flea 3) from the side and also from below (through the Perspex block) to observe the sample morphology and dynamics. The motion of the sample edge was also quantified using image analysis of the side view. A binary image of the sample was generated through thresholding (OpenCV, Python). From this the width of the sample at different heights could be calculated in each frame. The width very close to the rheometer plate and the minimum width in each frame, occurring at approximately the midpoint between the plates, were then extracted to quantify the sample slip and break-up behaviour. The gap between the plates was calculated by multiplying the time by the known plate velocity. The sample moisture content was stabilised throughout the experiment using transparent Perspex covers with in-built moisture reservoirs (humidity $\sim$ 90%). These were only briefly removed to load and trim the sample. Independent tests were carried out which showed that the mass loss over the timescale of the experiments ($\sim$ 20 min) was $< 0.5\%$. A few experiments were also carried out using rheometer plates covered with P40 and P320 sandpaper (grit size $\sim \, 350\,\upmu \hbox {m}$ and $45\,\upmu \hbox {m}$, respectively).

All samples were pre-sheared at $$20 \, {\hbox {s}}^{{-}1}$$ for 5 min at a gap height of 1.05 mm (except where specified) before trimming and lowering to a working height of 1 mm. Several different types of rheological measurements were made on the samples following the initialisation protocol. All the different rheological measurements were performed at least 3 times. Adhesion tests were performed by raising the upper plate at a constant vertical velocity of $$10 \, \upmu {\hbox {ms}}^{{-}1}$$ (except where specified), whilst measuring the normal force on the upper plate. The velocity was chosen to ensure the estimated shear flow at the sample edge was well below that required for bulk fluidisation (see discussion Sect. 3.5). The bulk properties of the samples were characterised in shear using the 20-mm diameter parallel plate geometry covered with P40 sandpaper. Shear start up measurements were performed at a constant shear rate of $$0.01\,{\hbox {s}}^{{-}1}$$ (comparable to the extensional rate $${{V}}_{{z}}/{{h}}_{0})$$. Stress against strain plots (see supplementary info) were used to extract the measured yield stress from the maximum stress value for all the concentrations used [25]. Selected concentrations were also measured using a decreasing shear ramp (1 decade/minute, $${\dot{\gamma }} \sim 20{-}10^{{-}3} {\hbox {s}}^{{-}1})$$. Amplitude sweeps in oscillatory shear were also performed at 0.4 Hz to measure the bulk visco-elastic moduli. Estimates of the yield stress with all of these techniques gave comparable values (see supplementary information). Prior to these measurements some samples were sprayed with a vertical line in red acrylic paint using a stencil to confirm there was no slip [34, 35]. Similar experiments were also performed with the roughened Perspex to observe the slip behaviour during the initial stages of the flow of 50 and 60 wt% clay. This technique was also used to confirm that the sample was fully fluidised during the pre-shear sample preparation protocol. Whilst our aim was to study the effects of slip, which is more pronounced on smoother surfaces, initial tests indicated that using Perspex plates sandblasted with a finer grit did not exhibit bulk fluidisation even at high shear rates. In contrast, the roughness of the Perspex plates used in this study allowed strong bulk fluidisation at high shear rates, ensuring a reproducible initial state. In between each set of experiments both Perspex surfaces were carefully cleaned to remove all clay and then dried with $${{N}}_{2}$$.

By default the normal force sensor on the rheometer uses a low pass frequency filter which is used to smooth the data. Unfortunately, this can result in increasing and decreasing changes in force being smoothed out if the cut off frequency is too low (i.e. the measured peak value is reduced). In order to be confident of the quantitative forces measured at the peak we switched the frequency filtering off and then after the measurement filtered the raw data using a low pass frequency filter. In doing this we checked carefully that the cut off value did not alter the shape of the measured curves.

## Results and discussion

### Concentration dependence of adhesion tests

**Fig. 1:**
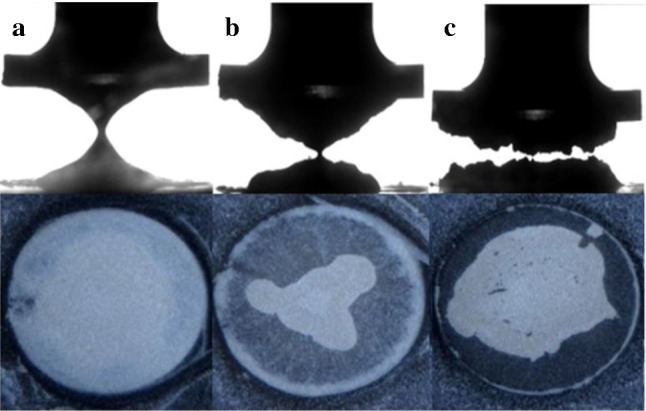
Images taken from the side and from below the kaolin samples during adhesion tests ($\hbox {velocity} = 10 \, \upmu {\hbox {ms}}^{{-}1}$) on the roughened Perspex plates, (a) 45 wt%, (b) 55 wt%, and (c) 65 wt%. The samples show a transition from filament to fracture-like breakup behaviour

Adhesion tests were carried out on kaolin suspensions at a range of concentrations between 45 and 65 wt% ($\phi \sim 0.31{-}0.47$). In each measurement the upper geometry was slowly raised at a constant velocity of $10 \, \upmu {\hbox {ms}}^{{-}1}$. Example movies of the lowest (45 wt%) and highest (65 wt%) concentrations are also shown in supplementary movie 1. In low concentration samples the middle of the sample progressively thins in a smooth fashion resulting in the deposition of two conical deposits (Fig. 1a). This is similar to the behaviour observed by a number of authors in the model fluid Carbopol [19, 27]. However, the break-up behaviour of high concentration samples is very different. These samples do not neck and flow as before but after moving a small distance towards the centre, the fluid breaks up by a solid-like fracture process (Fig. 1c). These dynamics strongly depend on the clay concentration with a gradual crossover in the behaviour observed at $\sim$ 50-55 wt% (Fig. 1b).

Observation of the adhesion test from below the sample, via the transparent bottom plate, reveals some interesting differences in the deposition behaviour as the concentration of the suspension is increased (see Fig. 1 lower panels). Each image shows the final configuration of deposited fluid after breakup. In the middle of each image a white section shows the bulk clay, which is thick enough to be opaque. At the periphery of the sample the highest concentration clay shows a thin white bead of clay. This is wider at intermediate clay concentrations and appears to extend from the edge all the way to the centre for the lowest concentrations. In between the periphery and the central mass of clay we observe that a sparse thin film of clay is left behind on the surface. This region corresponds to the moving contact line of the samples. At the low clay concentrations this film is significant and almost thick enough to be opaque. In contrast at the highest concentration there is almost no clay adhered to the surface in this region. The central region of deposition which is conical and relatively symmetric at low concentrations becomes quite unstable and asymmetric with something a bit like a fracture surface at the highest concentrations [17]; however we do not observe the fractal-like structures that have been observed in other yield stress fluids [15, 19–21]. This is perhaps due to the higher yield stress of our paste, a property which can suppress fingering [19]

**Fig. 2:**
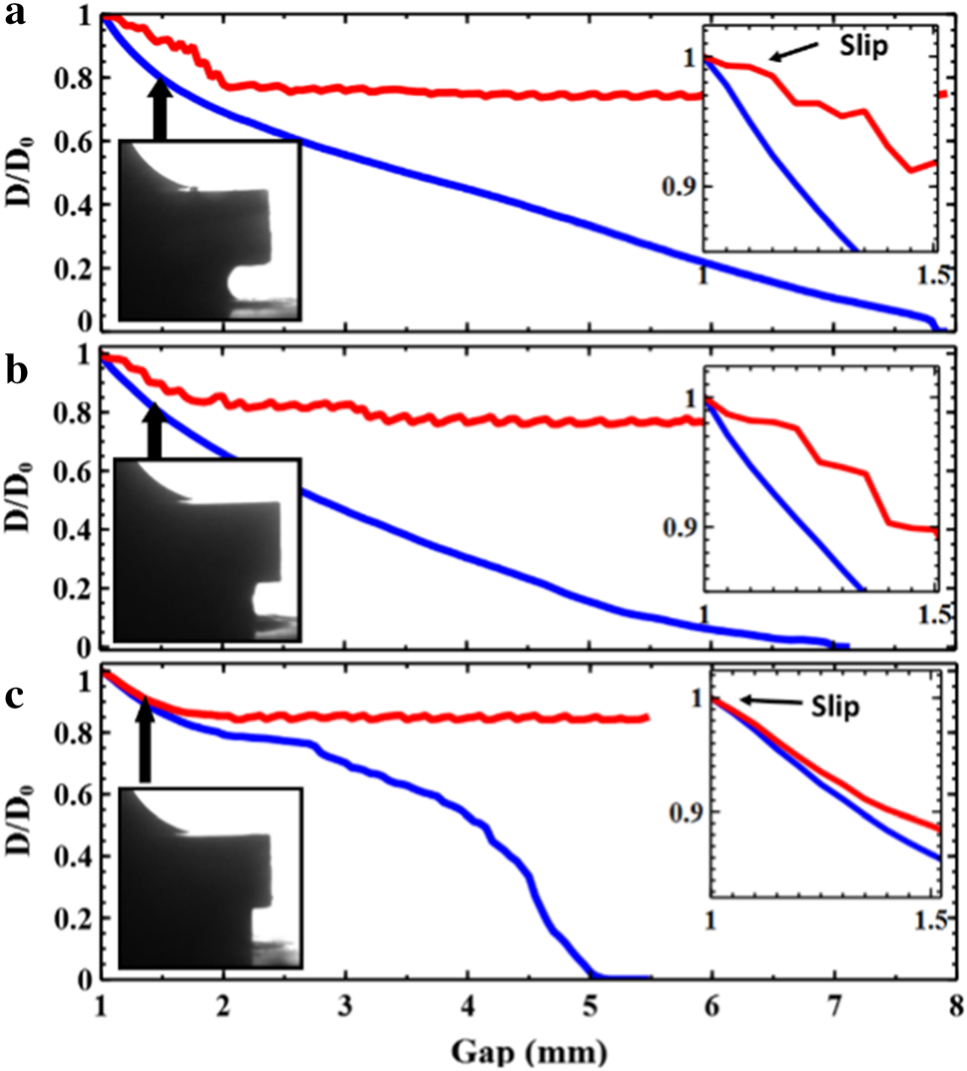
Changes in width with rheometer plate gap of (a) 45 wt%, (b) 55 wt% and (c) 65 wt% Kaolin clay during adhesion tests. In each plot the blue line represents the minimum width of the sample which occurs approximately at the midpoint between the upper and lower rheometer plates. The red line represents the width of the clay just above the bottom plate ($\sim$ 0.05 mm). The insets show in more detail the dynamics of the early slip behaviour. A representative image is also shown for the early stages of each experiment. The high concentration samples initially slip without deformation of the edge, whereas low concentration samples initially pin and deform before slipping

One feature immediately apparent from videos of the different concentration experiments is that the initial stages, for both low and high concentrations, look qualitatively different, in each case exhibiting significant slip. These qualitative observations of the dynamics can be confirmed more precisely (Fig. 2) by measuring the width of the sample (viewed from the side) as a function of the plate separation (see methods). We measure near the rheometer plate (red) and at the narrowest point (blue), which occurs near the middle of the sample. The width measurements in Fig. 2 close to the plate (red lines) show some small oscillations. These are an artefact of the image processing due to small variations in lighting and the acute contact angle at the edge of the sample. That this is the case can be seen from the fact that they persist even once the contact line remains stationary. Despite this the differences in sample dynamics are clear.

Figure 2a shows a 45 wt% sample. From the very first moments of the experiment the width decreases more quickly at the midpoint than near the plate, as a consequence of a pronounced curvature developing at the sample edge. As the plate continues to rise, the contact line undergoes apparent slip. When the contact line has moved approximately a quarter of the way from the edge to the centre it then stops moving. The central region then continues to narrow at a constant rate. Finally, the thin neck between the two halves breaks up, leaving two conical deposits behind.

In contrast to this, the high concentration samples exhibit very different dynamics (see Fig. 2c). At the beginning of the experiment both the mid-point and the region near the plate move together (see inset Fig. 2c). This corresponds to an initially smooth and approximately vertical contact line slipping along the bottom plate deforming as a block. There then follows a small region in which the sample appears to neck slightly ($\hbox {Gap} = 1.5{-}2\hbox {mm}$). However, this is quickly overtaken by a break-up process that resembles fracture, in which the sample does not slip.

Although the analysis here helps to clearly delineate the different sample dynamics, it is difficult to reliably extract quantitative information (e.g. contact line velocities) from these plots. As seen in Fig. 1d–f the final sample morphologies can be at times quite asymmetric. Consequently, the widths measured would depend on the orientation of the sample relative to the camera.

**Fig. 3:**
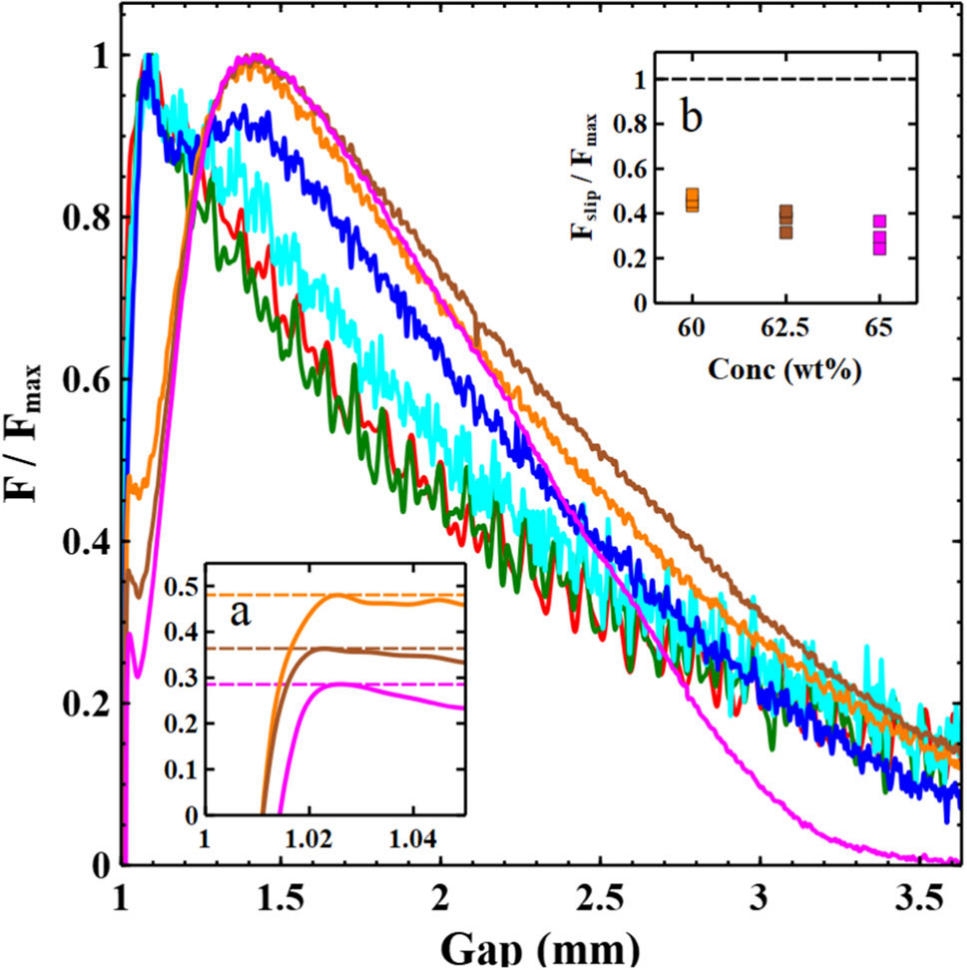
${{F/F}}_{\mathrm {max}}$ for samples of different clay concentration. 45 wt% (red), 47.5 wt% (green), 50 wt% (blue), 55 wt% (turquoise), 60 wt% (orange), 62.5 wt% (brown), 65 wt% (pink). The curves fall into two different qualitative shapes which correlate with the initial slip behaviour at the edge of the sample. Insets: (a) The initial “kinks” in the force data for the high concentration samples which correspond to the onset of slip, the dotted lines indicate the definition of ${{F}}_{\mathrm {slip}}$. (b) A plot showing how the ratio ${{F}}_{\mathrm {slip}}/{{F}}_{\mathrm {max}}$ varies with sample concentration

**Fig. 4:**
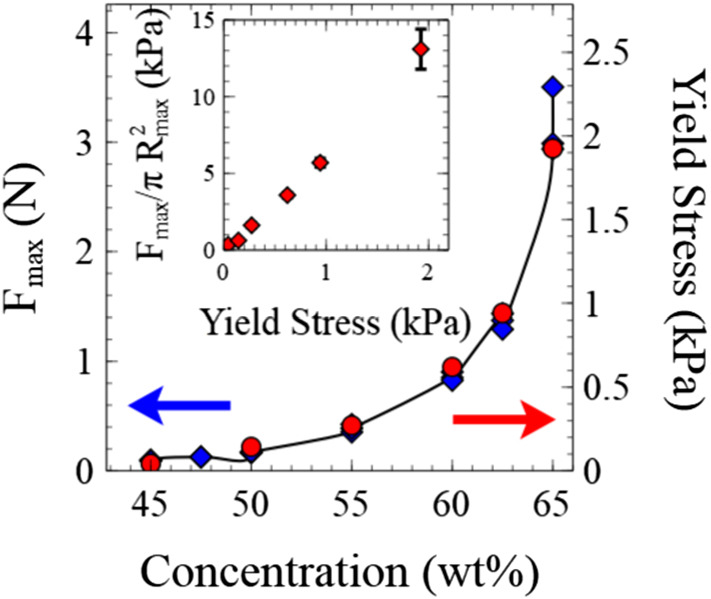
Comparison of the maximum measured force during adhesion tests (blue) with the measured sample shear yield stresses (red) as a function of sample concentration. The inset shows the approximately linear relationship between yield stress and the scaled maximum force. The force has been scaled by an estimate of the approximate area of the sample at which ${{F}}_{\mathrm{max}}$ was measured which is slightly different for low and high concentration samples

Figure 3 shows the normal force curves for different clay concentrations, divided by the maximum measured force (${{F}}_{\mathrm {max}})$. Measurements of the shear yield stress were also obtained using the same parallel plates covered in P40 sandpaper, which prevents slip (see methods). The maximum normal forces measured during the different experimental adhesion tests mirror these large changes in material stiffness spanning two orders of magnitude (see Fig. 4). The adhesion test force curves clearly fall into two qualitatively different categories of behaviour. The differences in concentration profoundly affect the shape of the early stages of the measured curves.

The initial rise in the force during an adhesion test has commonly been attributed to some sort of elastic or viscoelastic loading of the sample [10]. All our samples exhibit an initial steep increase in force with separation which is consistent with this. Under some experimental conditions the initial rise in force has also been attributed to the compliance of the rheometer/normal force sensor [15, 18]. One can assess the likely impact of this on our experiments by measuring the spring constant of the rheometer load cell. To measure the stiffness of the sensor we placed a steel disc on the rheometer and applied increasing levels of compressive force with the upper plate. Measuring the recorded displacement gives a straight line with gradient $412.3 \pm 0.2 \, {\hbox {kNm}}^{{-}1}$ equal to the effective spring constant of the normal force sensor (see supplementary information). This is an order of magnitude larger than the biggest force gradient measured in this study corresponding to a $\sim 2.5\, \upmu \hbox {m}$ gap correction for a 1N load making this at most a small correction. Furthermore, the motion of the plates is always slow, such that inertia can be neglected. We therefore conclude that the rise in force simply reflects sample properties.

In the low concentration samples the force curves have a relatively simple form. The force rises to a maximum, after a small movement of the plates (${{h/h}}_{0} \sim 1.08$), and then rapidly decays. The slip of the contact line starts at the position shown in the inset of Fig. 2a (${{h/h}}_{0} \sim 1.1 \pm 0.025$). The onset of sample slip and the maximum in the force curve appear to occur together for low concentration samples. An initially pinned contact line, with visco-elastic loading of the bulk sample results in a critical stress that is sufficient to produce yielding of the sample. As the sample contact line begins to slide, the deposition of a substantial layer of clay on the surface (see Fig. 1d) seems to indicate that the slip occurs by a kind of shear localisation, resulting in the strong yielding of a thin layer of material near to the plates.

**Fig. 5:**
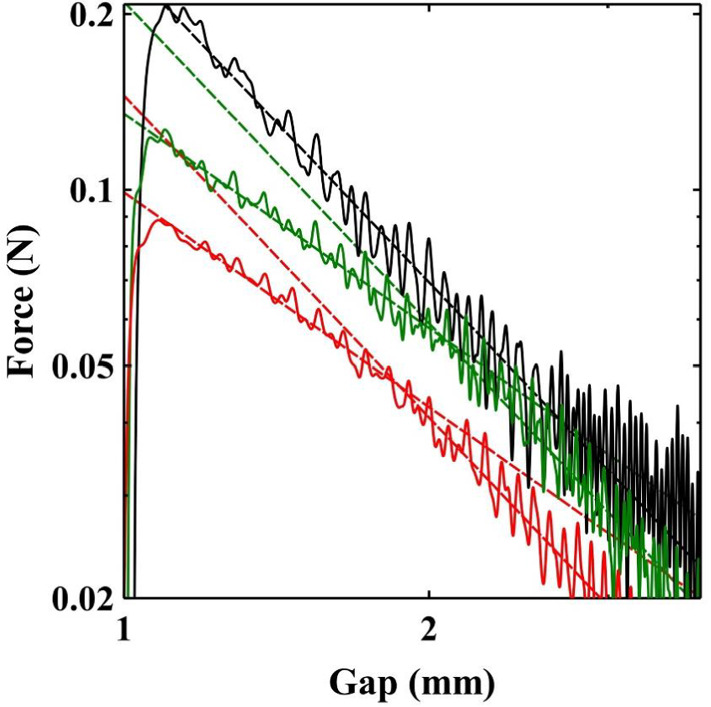
Force distance curves for a 2 wt% Carbopol suspension (black) and the lower concentration samples: 45 wt% (red), 47.5 wt% (green). The Carbopol suspension which does not exhibit slip has a gradient on a log–log plot of $-\,1.9$. In contrast the low concentration kaolin samples, following the maximum in the measured force, slip resulting in a gradient of $-\,1.2$. As the plates continue to separate the sample stops slipping which corresponds to a change in the measured gradient to $\sim \, -\,1.9$

Following this maximum the measured forces decay as the sample yields and slips concurrently. Figure 5 shows a log–log plot of the force against separation for the low concentration samples. The scaling of the force with plate separation in an adhesion test is known to depend on the boundary conditions at the interface. Samples undergoing perfect slip exhibit an extensional flow with a force that scales with ${{h}}^{{-}1}$ [23]. For comparison we provide a similar measurement obtained in the same way for a 2 wt%, Ultrez U10 Carbopol suspension (Black) which behaves as a model simple yield stress fluid and has been frequently studied with adhesion tests [19, 23]. The concentration of Carbopol was chosen to give force measurements of similar magnitude to the low concentration clay samples. Whilst our data covers a limited range and is somewhat noisy it is possible to extract the power law exponents. A fit to the Carbopol data (black), after the peak, results in a power law exponent of $\sim \,-\,1.9$ for nearly the entire range of the data. Observation of the Carbopol sample reveals very little motion of the contact line/slip. In this and similar measurements by other authors [15, 19] the exponent remains constant until the final stages of breakup. In the low concentration clay samples we however observe that the power law exponent is initially much shallower $\sim \, -\,1.2$ and then transitions to a gradient of $\sim \, -\,1.9$ (see Fig. 5). Divoux et al. [21] studying a particle gel, also observed a change in the power law exponent during an adhesion test to a value $\sim \,-\,2$. The transition to the second stage occurred at a critical gap at which the final deposit diameter remained constant. The exponent -2 was found to be related to the necking behaviour of the filament. Figure 5 shows that the transition in our samples occurs at a gap $\sim$ 2mm. Comparing this with the width measurements in Fig. 2a, it seems that this is also the gap at which the contact line is arrested. As the experiment proceeds the aspect ratio of the sample (h/R) grows. The difference in cross-sectional area between the midpoint and the end plates also increases. Consequently, the stress is largest at the midpoint and reduces towards the boundaries. Beyond some critical separation, the stress required at the boundary to create slip is no longer exceeded. It seems therefore that the change in exponent reflects the crossover from a force curve that is strongly modified by slip, to one in which the forces reflect solely the necking of the bulk material.

At high clay concentrations we observe a slightly more complicated shape of force curve. The force undergoes an initial steep linear rise (see Fig. 2 main panel and inset c). This initial rise however has a “kink” at very small strain values (${h/h0} \sim 1.025$, see inset a, Fig. 3). Figure 2c shows images of the edge of the 65 wt% sample. This, unlike the low concentration samples, appears to slip almost immediately with a contact angle of close to $90^{\circ }$. In these stiffer samples, the normal stress for a given extensional strain is larger, exceeding the stress required to induce slip before significant deformation of the exposed interface has begun. The sample therefore begins to slip along the plates, deforming as a cylindrical block, until a plate separation $\sim 1.25 \pm 0.025 \, \hbox {mm}$. At this point there is a difference for the first time in the narrowing of the contact line and the sample midpoint. The contact line now gradually slows before becoming completely pinned at ${{h/h}}_{0} \sim 1.8$. The onset of the “fracture-like” break up begins prior to the cessation of motion of the contact line but after the midpoint has begun to narrow faster than the contact line. The maximum in the force curve occurs between these two points at ${{h/h}}_{0} \sim 1.4$, although unlike the low concentration samples it is a broad peak and does not seem to be linked to a specific event, as the onset of slip was for the kink.

The influence of the very initial motion of the contact line on the shape of an adhesion test’s force–distance curve was commented on previously by Zhang et al. [23] in a slightly different context. They found that with the same sample conditions and experimental parameters there were significant difficulties in reproducing experimentally measured force curves. The curves exhibited two very different shapes and they explained this by the adherence or slip of the contact line in the initial phase. Adherence of the contact line would alter the local shear flow which then evolves as the experiment progresses. They postulate that in their experiments contact line adherence might be affected by drying or imperfections of the solid surface. In addition to using humidity covers (see methods) to prevent evaporative losses, we confirmed that drying was not responsible for the observed behaviour by performing experiments with a 20 min gap between trimming and the measurement. Adhesion tests performed with and without the wait had the same behaviour, indicating that drying of the sample periphery was not responsible. The roughened surface provides a large number of pinning sites. However, our experiments suggest that different concentration clays interact with this roughness differently, resulting in different slip conditions. The principal idea, that the initial adherence of the contact line plays a role in determining the shape of the measured curves, is however the same.

The measured force at which these samples begin to slip (${{F}}_{\mathrm {slip}}$) increases monotonically with the clay concentration. Figure 3, inset b shows that the ratio ${{F}}_{\mathrm {slip}}/{{F}}_{\mathrm {max}}$ of the higher concentration samples decreases with increasing clay concentration. This indicates that relative to the stress generated by extension of the clay (which is a function of sample stiffness) it becomes easier for these samples to slip. Our observations in Fig. 1 of the deposited clay thickness indicate that as the concentration of clay increases the shear becomes increasingly localised at the boundary. Decreases in slip-layer thickness with increasing concentration of particles have been measured before in capillary flows but with slip-layers a fraction of a particle diameter [27]. The use of roughened surfaces in our experiments would make the development of a sub-particle diameter slip-layer impossible and mean this slip-layer must be thicker. Indeed, capillary flow of a concentrated hard sphere suspension comparing flow of smooth and rough surfaces showed a similar increase in the sheared layer thickness [36]. The interaction of the particles with the rough surface topography is complex with effects such as particle layering and jamming. However, it appears that the thickness of the surface slip layer still decreases with particle concentration and is correlated with an increased propensity for slip prior to strong yielding.

Following this initial rise, the force in the high concentration samples continues to increase to a maximum. Figure 3 shows that the higher concentration samples have a maximum in the force at a significantly larger plate separation (${h/h0} \sim 1.4$) than the low concentration samples. This is long after the sample has begun to slip along the wall and seems to correspond to the first signs of the contact line becoming unstable and beginning to pin. An important consequence of the initial slip is therefore to delay the strong yielding of the bulk sample. After this one observes a fracture-like breakup. The crossover from a more ductile to more brittle-like fracture has been observed in similar experiments of both concentrated suspensions and foams and is likely related to the concentration dependent properties of the bulk. [37, 38].

### Relating the initial slip behaviour to observations in simple shear

Using a simple shear measurement at a constant shear rate of $0.01{\hbox {s}}^{{-}1}$ we observed the flow/slip characteristics for the 50 and 60 wt% clay samples on the same roughened Perspex plates. Our aim was to compare the transient behaviour at start-up, since this is similar to the situation encountered during the earliest stages of an adhesion test, where because of the small aspect ratio (${{h}}_{0}/{{R}}_{0}$) the flow may exhibit a significant shear component. A vertical line sprayed on the sample periphery with a stencil enabled us to observe how the clay flows / slips [34, 35].

**Fig. 6:**
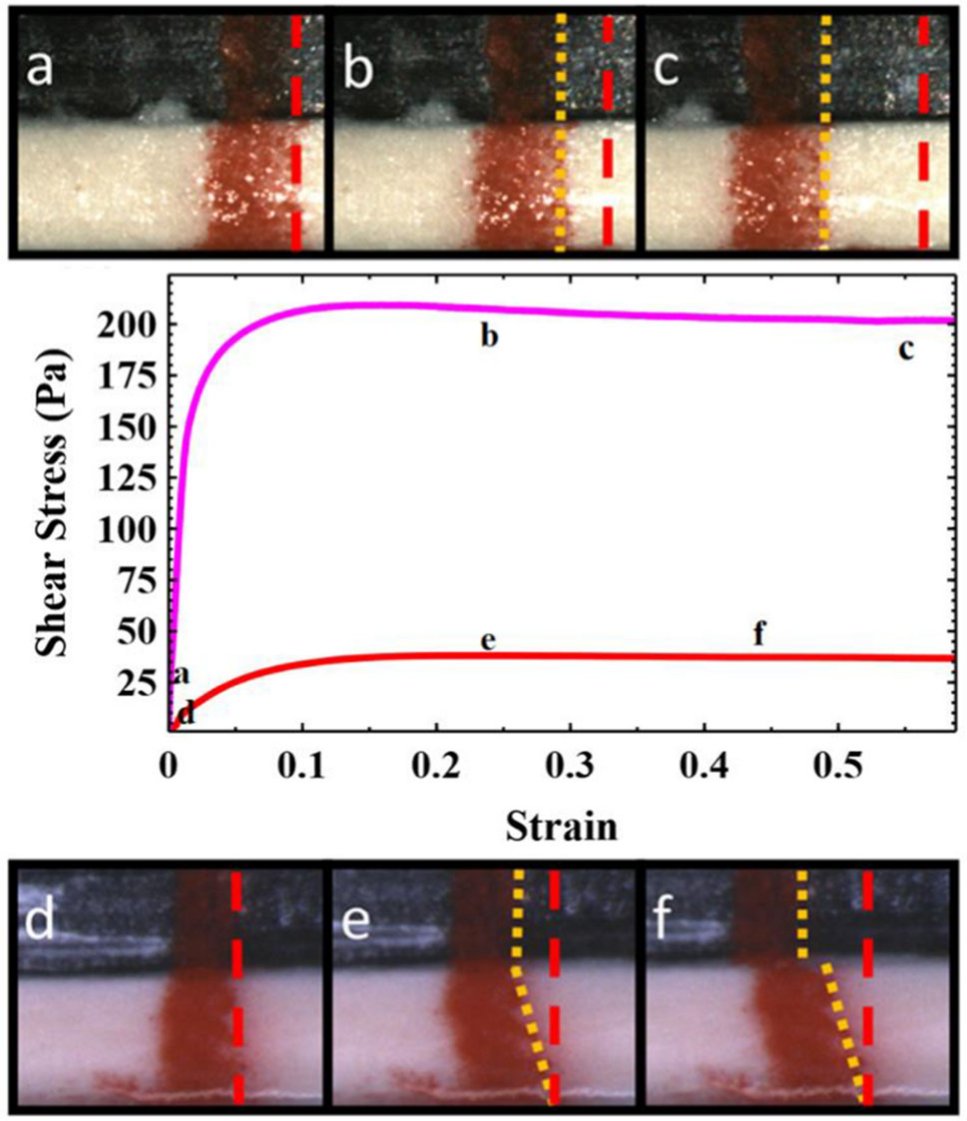
Observing sample deformation and slip in the start-up of simple shear experiments for a 60 wt% (a,b,c, pink) and 50 wt% (**d**,**e**,**f**, red) kaolin clay at a shear rate of $0.01{\hbox {s}}^{-1}$. Before each shear start up experiment a red line is sprayed vertically, coating the outside edge of the rheometer plate (black) and the clay (white). The superimposed red line shows the initial position of the right hand edge of this line. The superimposed yellow line tracks this same edge throughout the experiment. The higher concentration sample (top) undergoes no observable deformation, and slips relative to the bottom plate like a plug almost immediately (**b**,**c**). In contrast the lower concentration sample (bottom) initially does not slip and undergoes a shear deformation (**e**). When the sample reaches a critical strain value $\sim$ 0.25 the sample then begins to slip relative to the top plate (**f**), with the bulk undergoing no further deformation

Figure 6 shows example images showing the sample deformation at the appropriate points on the stress–strain curve. The stress–strain curves in both cases plateau at lower values than those measured with P40 sand paper in the absence of slip (see Fig. 4 and supplementary information). The plateau stress here, whilst still related to the sample yield stress, is reduced by the slip. Prior to reaching steady-state slip, the two samples start-up behaviour is apparently quite different. For the higher concentration sample the fluid apparently undergoes very little deformation before the sample slips cleanly at one interface. In contrast for the low concentration samples we initially observe no slip. A constant velocity gradient results in the line initially rotating with the shear profile. When the shear strain reaches a critical value $\sim$ 0.25 the sample then begins to slip along the moving plate.

This experiment mirrors some of the observations on slip deduced from our adhesion tests, supporting our interpretation. There is a change in the slip behaviour that results in slip with very little sample deformation at high concentrations and a strong deformation of the sample prior to slip at lower concentrations. However, we did also notice some differences between the two types of experiment. In the adhesion test at low concentrations, a thick film of particles is left behind as the clay yields and slips. One might therefore expect an equivalent thin shear band near one of the plates in shear. It is possible that it is too thin to observe, but we estimate this should be within the resolution of our images. Another noticeable feature in the shear experiments is that once the lower concentration sample begins to slip there is no further deformation/yielding of the bulk clay. This is clearly seen by the fact that the red line undergoes no further deformation. This is in contrast to the adhesion test where the sample edge deforms up to the point at which slip occurs, thereafter strongly yielding. We believe it is likely that these two observations are related. In the simple shear experiment the shear stress is constant throughout the thickness of the sample. In contrast the deformation of the interface at the edge of the low concentration adhesion tests results in a narrowing of the central portion of the sample. There is therefore a lower stress at the plate boundaries than at the midpoint. This might suggest that the deposited material is the result of the stress gradient induced by the initial pinning and deformation of the contact line. That we see far less material deposited by higher concentration samples would fit with this interpretation since the vertical contact line ensures a relatively uniform stress.

### The effect of surface roughness

Our initial experiments raise some interesting questions as to what extent and in what ways the initial slip characteristics influence the final breakup behaviour and measured force curve? To test this we performed adhesion tests using a P40 and P320 sandpaper (grit sizes $\sim 350\, \upmu \hbox {m}$ and $\sim 50\, \upmu \hbox {m}$). In constant shear-rate experiments, using the P40 sandpaper, slip was found to be completely suppressed. In adhesion tests the sample still underwent some slip across the sample surface but it is clear that the slip is significantly reduced. The 60 wt% sample on the Perspex maintains a very straight edge as it slips, only deviating once the plate gap ${h} \sim 2.5\,{{h}}_{0}$. In contrast, on the P40, the clay in contact with the plates initially pins and then lags behind the mid-point of the sample before again pinning and undergoing breakup by a horizontal fracture-like process. Whilst the slip characteristics on the rougher surface are similar to lower concentration samples, the final breakup behaviour still undergoes a fracture-like process consistent with the high concentration clay.

**Fig. 7:**
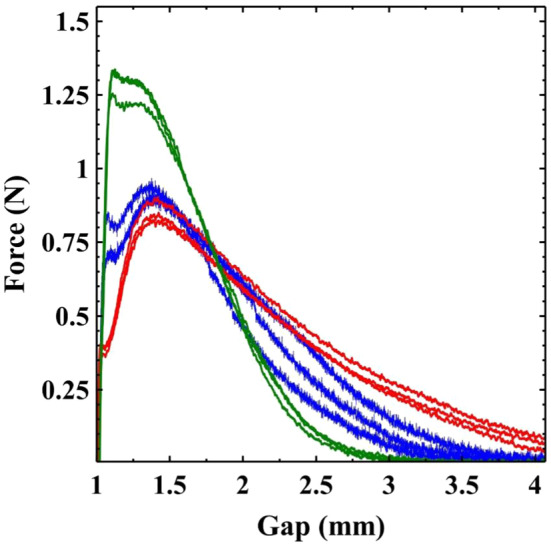
The effect of surface roughness on adhesion tests of a 60 wt% Kaolin paste. Data is shown for measurements on the roughened Perspex (red), P40 sand paper (green, grit size $\sim 350\, \upmu \hbox {m}$) and P320 sand paper (blue, grit size $\sim 50\, \upmu \hbox {m}$)

Figure 7 shows 3 repeat adhesion test measurements of a 60 wt% Kaolin clay for the roughened Perspex (red) and the much rougher P40 (green). We see a considerable change in the qualitative shape of the measured force curves. To confirm that this was caused by the increase in surface roughness rather than the chemical properties of the surface we also used a finer sand paper P320 (blue) which more closely matched the Perspex than the rougher P40. Comparing the curve shapes with our concentration dependent measurements the rougher surface appears to have the qualitative shape of a lower concentration sample. Earlier we argued that it was harder (relatively speaking) for the low concentration samples to slip. Increasing the roughness of the surface also makes it harder for the samples to slip. Divoux et al [26] showed in simple shear start-up experiments on a microgel that reducing the surface roughness decreased the maximum measured stress and critical strain at which slip began. Our adhesion tests exhibit this same behaviour with smoother surfaces slipping at smaller strains and lower forces. We also invariably observe a small drop in force at the moment slip begins, before the measured force continues to rise. This relaxation of elastically stored stress may be akin to the stress overshoot sometimes observed during transient start-up measurements which can be related to slip [24, 26]. As noted the final observed breakup behaviour was not so strongly influenced by the change in surface roughness. The fact that this is not obvious from the force curves is due to two factors. Firstly, breakup occurs when ${{h/h}}_{0} > 3$ by which time the cross-sectional area of the sample and hence measured forces are much lower (cf Fig. 2). Secondly, in all cases the sample stops slipping prior to final breakup at which point the breakup behaviour must be solely determined by the sample’s bulk properties.

### Modifying the initial compressive normal force

Our measurements indicate that the initial development of slip in our experiments can subsequently influence the dynamics and breakup behaviour during an adhesion test. The development of slip during the start of the flow involves the initial interactions of the particles with the confining surfaces and each other. This is presumably modified by factors such as how well the constituent particles pack into the topography of the roughened surface, which generates resistance to slippage [28]. Any changes to this packing not only modify the local volume fraction but as a consequence the amount of water, which lubricates particle–particle and particle–surface contacts.

**Fig. 8:**
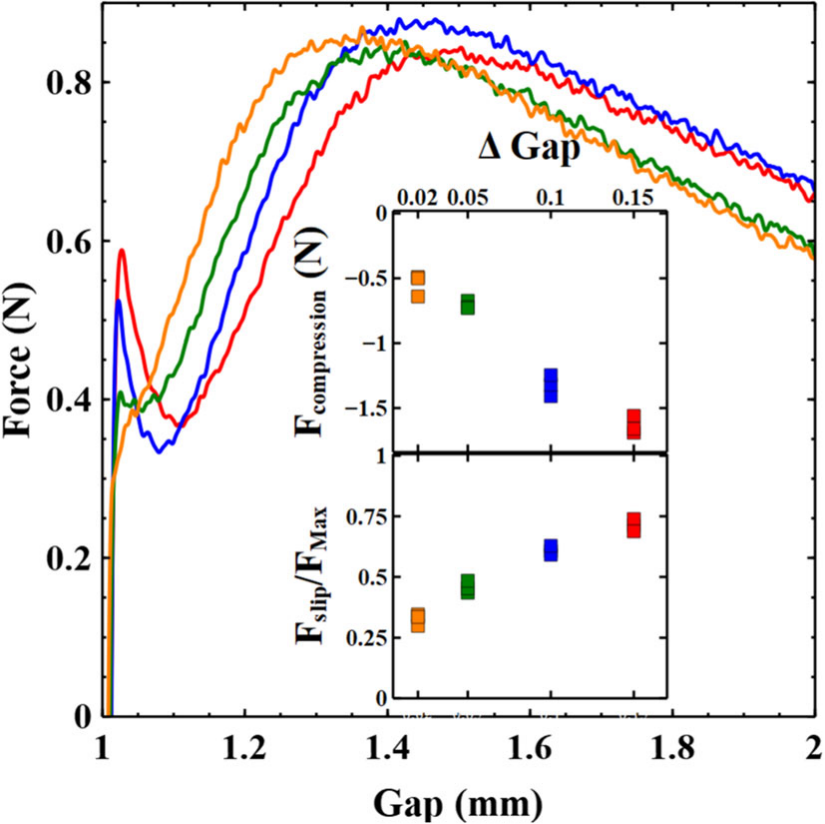
Changes in slip as a function of the sample protocol’s compressive load. Samples of 60 wt% kaolin are pre-sheared at slightly different initial gap heights and then trimmed. The gap height is then lowered by $\Delta$ Gap (mm) to 1mm. Different compression steps result in a different compressive load. Following this the same adhesion test is performed, moving the plates apart at $10 \, \upmu {\hbox {ms}}^{-1}$. The value of ${{F}}_{\mathrm{slip}}$ indicated by the initial “kink” changes as the compressive load increases. The upper inset shows the dependence of compressive load on the compression step size. The lower inset shows how the value of ${{F}}_{\mathrm{slip}}/{{F}}_{\mathrm{max}}$ is modified by the initial compressive load. It indicates that there is an increased resistance to slip following an increase in initial compressive load during the preparation protocol. The colours in both insets and main panel correspond to the same changes $\Delta$ Gap as indicated in the inset

We performed a series of adhesion tests with 60 wt% clay in which we slightly modified the preparation protocol. The pre-shear of the clay was performed at slightly different gap heights ranging from 1.02–1.15mm. At the end of the pre-shear the normal force measured is zero. Following the pre-shear the samples were trimmed and then the plate lowered to the same initial gap height of 1mm. Since each sample undergoes a different amount of compression the sample is subjected to a different final compressive normal force which depends on the clay concentration and the change in gap height prior to the experiment (upper inset Fig. 8). A particularly interesting observation during this approach to the initial height is that one can observe a small amount of clear fluid escaping at the periphery of the samples near to the confining rheometer plates during the larger compression steps. Supplementary video 2 shows an example of this with a 60 wt% clay undergoing the compression step prior to the adhesion test.

Following this difference in preparation history we perform identical adhesion tests, that is the samples have the same initial gap height (1mm) and the upper plate is moved upwards at the same speed ($10\, \upmu {\hbox {ms}}^{{-}1}$). Figure 8 shows how the forces measured in the early stages depend on the separation of the plates. A significant increase in the value of ${{F}}_{\mathrm {slip}}$ with increased compression is observed indicating that the initial resistance to slip is significantly increased. However, as soon as slip begins it is obvious (particularly in the more highly compressed samples) that there is a relaxation of the force before it again builds to the maximum. This would seem to indicate that the samples initially undergo visco-elastic loading up to a maximum determined by the force required to induce slip. Slip then releases some of this stored energy before stresses again build up in the sample. Before examining concerns about some of the potential difficulties in interpreting these experiments it should be noted that at the very least this provides a simple way to modify the initial slip characteristics of a paste without having to alter the sample or surface topography.

There are however potential difficulties with these experiments, which require careful consideration. Firstly, it is clear that there will be a slight under-/over-filling of the sample edge. The majority of experiments in this paper were performed using a change in gap height (between trimming and the starting gap height) of 0.05mm. This value was chosen since it resulted in an initially square edge to the sample. Assuming a constant sample volume and that the sample deforms as a cylinder, we estimate that the sample radius would therefore be 0.25 mm (2.5%) overfilled for $\Delta \hbox {gap} = 0.1\,\hbox {mm}$ and 0.15 mm (1.5%) underfilled for the $\Delta \hbox {gap} = 0.02\,\hbox {mm}$. Secondly, the increased normal force may result in some compaction of the clay, which may or may not be reversible. The escape of water from the sample indicates that there must be some compaction. However, the water appears to be escaping near the plates, and there are no obvious changes to the sample surface at the midpoint (see supplementary video 2). The additional volume amounts to just a few per cent but it could have an influence on what is measured. For example, the gap at which ${{F}}_{\mathrm {max}}$ is measured seems to increase with larger compression steps. It would make sense that if there is slightly more clay, that the gap at which different features are observed increases.

Whilst some compaction occurs, one might expect this to make the measured force curves appear more like those of higher concentration samples. Ideally one would like to measure the samples bulk viscoelastic properties following each compressive step. We attempted to perform oscillatory measurements of the moduli; however, due to the slip relative to the rheometer plates this resulted in very unstable measurements which weren’t reproducible. The normal force measurements do however show that ${{F}}_{\mathrm {max}}$ is independent of the initial compression (Fig. 8). In Fig. 4 the value of ${{F}}_{\mathrm {max}}$ was shown to be strongly correlated with the bulk yield stress of the sample, providing some evidence that the bulk properties are not significantly altered. In fact after the initial slip, the shape of the curves looks very similar (though slightly shifted). In light of this it’s interesting that there is such a dramatic increase in ${{F}}_{\mathrm {slip}}$ with initial load. Figure 8, lower inset, shows the ratio ${{F}}_{\mathrm {slip}}/{{F}}_{\mathrm {max}}$ for these samples. ${{F}}_{\mathrm {slip}}/{{F}}_{\mathrm {max}}$ increases with an increase in the initial compression step. Comparing this data with inset b shown in Fig. 3 also indicates that with increasing compression of the sample the slip characteristics are actually more like those of lower concentration samples. This is the opposite of what one might expect if the changes in slip were simply due to compaction of the clay during the compression step. The increase in ${{F}}_{\mathrm {slip}}$ would however make sense if the clay particles are compressed into a more tightly packed/jammed structure in the rough surface topography. This would also explain why water is seen to emerge preferentially at the surface of the rheometer plates. The increased inter-particle friction and interactions with the surface would make it harder for the sample to generate a slip layer by yielding locally, whilst leaving the bulk properties largely unchanged.

### The effect of strain-rate on observed slip characteristics

**Fig. 9:**
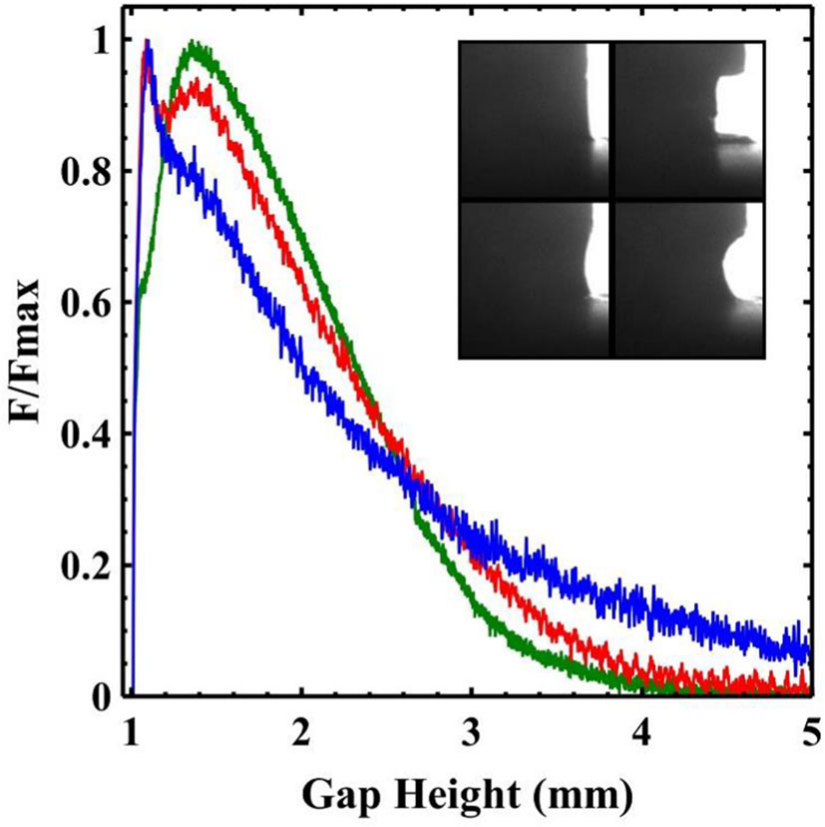
Adhesion test force curves for 55 wt% Kaolin paste. Curves are shown for plate velocities of $100 \, \upmu {\hbox {ms}}^{-1}$ (blue), $10 \, \upmu {\hbox {ms}}^{-1}$ (red), $3.3 \, \upmu {\hbox {ms}}^{-1}$ (green). Inset: images of the sample periphery for the lowest strain-rate (top) and highest strain-rate (bottom)

To explore the dependence of strain-rate on slip, we focussed on the intermediate clay concentration of 55 wt%. Figure 9 shows 3 sets of adhesion tests conducted with the 55 wt% clay samples. In these measurements we altered the plate velocity to see whether the shape of the measured force curves indicated changes in slip behaviour. Data is shown for measurements obtained with ${{V}}_{{z}} = 100$, 10 and $3.3\, \upmu {\hbox {ms}}^{{-}1}$. The lower velocity was chosen such that an experiment could be completed inside 20 min (the timescale for which we previously checked the results were unaffected by drying). Shear measurements of the 55 wt% sample showed that the measured stress did not increase significantly above the yield stress at shear rates below $2 \, {\hbox {s}}^{{-}1}$. We therefore selected $100 \, \upmu {\hbox {ms}}^{{-}1}$ on the basis that with an aspect ratio ${{h}}_{0}/{{R}}_{0} = 0.1$ a crude estimate of the apparent shear-rate induced at the edge of an adhesion test (for a completely pinned sample) would be ${{2v}}_{\mathrm {radial}}/{{h}}_{0} \sim 2{{R}}_{0} {{V}}_{{z}}/{{h}}_{0}^{2} = 2 \, {\hbox {s}}^{{-}1}$. Measurements of the work of adhesion showed no appreciable change as a result of rate, indicating that the viscous term associated with the adhesion energy still scales simply with the yield stress [19] as it did for our earlier measurements (Fig. 3).

At the lowest plate velocity one observes that the characteristic shape of the force curves changes to more closely resemble that of the high concentration clays with a single peak at ${h/h0} \sim 1.4$. We see a vertical contact line that slips smoothly across the surface. As the plate velocity is increased the corresponding force curves begin to more closely resemble the lower concentration clays. The force curve at the lowest plate velocity has a marked kink at ${F} \sim 0.6$ indicating the onset of slip significantly before strong yielding at ${{h/h}}_{0} = 1.4$. The force curve for the highest plate velocity does not quite have the same shape as our lowest concentration samples. There is still a significant shoulder at ${h} \sim 1.3 \, \hbox {mm}$ in a similar manner to the 50 wt% sample in Fig. 2. This indicates that the slip behaviour should be somewhat intermediate between the extremes illustrated in Fig. 2. The sample periphery for the highest rate experiment does appear to have more curvature than the lowest rate, and the slip behaviour is likewise altered in the ways previously outlined.

Only a few studies have looked at the rate dependence of slip during transient rheological experiments for yield stress fluids. LAOS experiments on a PDMS yield stress fluid found no dependence on shear-rate [39]. Divoux et al found that shear-rate did modify the measured stress overshoot and slip behaviour of a colloidal gel. However, this dependence appears to relate to the changes in sample elastic modulus with waiting time [26]. In our concentration dependence studies we noted changes to the thickness of deposited clay, thought to be associated with the slip layer. With our strain-rate dependent measurements, the changes to slip were more modest and hence it was difficult to confidently state whether more clay was or was not being deposited in each case. However, shear rate experiments below a critical value often lead to localised shear with a slip-layer thickness that depends on the slip velocity/apparent shear-rate [32]. Given our earlier observations it seems reasonable that there would be some associated change in slip layer thickness. Aral et al showed that the development of a slip layer to its equilibrium thickness can also require considerable time for a concentrated suspension [40]. One possibility is that the shear-rate dependence results from changes to the magnitude of ${{F}}_{\mathrm {slip}}$ that arise from the transient development of the slip-layer in the initial stages. However, more quantitative measurements of the growth of this slip layer during start-up would be required to clarify with confidence the origin of the shear-rate dependence.

## Conclusions

We have shown that whilst the breakup behaviour of a concentrated kaolin clay is controlled by the sample concentration, the initial stages of an adhesion test are very sensitive to slip. Changes in concentration, surface roughness, initial compressive load and extension rate all modify the slip conditions leading to two qualitatively different classes of force distance curve shape. As the force required for slip relative to the sample yield stress decreases, the plate separation at which the maximum force is measured increases. A small peak indicating the onset of slip, and the maximum at which large scale yielding of the sample begins become separated from one another. The changes in slip appear to originate from differences in the way the layers of clay near the roughened topology of the surface yields compared to that in the bulk. We also showed that the lubrication conditions near to the interface may be changed during the initial sample loading, potentially altering the subsequent slip behaviour.

## Supplementary Information

Below is the link to the electronic supplementary material.

Supplementary material 1 (pdf 417 KB)

Supplementary material 2 (m4v 898 KB)

Supplementary material 3 (mp4 3943 KB)

## Data Availability

The datasets generated during and/or analysed during the current study are available from the corresponding author on reasonable request.
