# Supplementary material for: Slip in adhesion tests of a Kaolin clay
Source: Eur Phys J E Soft Matter. 2021 Aug 11;44(8):102. doi: 10.1140/epje/s10189-021-00107-9 (PMC8357682; doi:10.1140/epje/s10189-021-00107-9)
Supplement: Supplementary file 1 — Supplementary material 1 (pdf 417 KB) [file 10189_2021_107_MOESM1_ESM.pdf]

# Supplementary information - Slip in adhesion tests of a Kaolin clay

M.J. Hayes, M.I. Smith

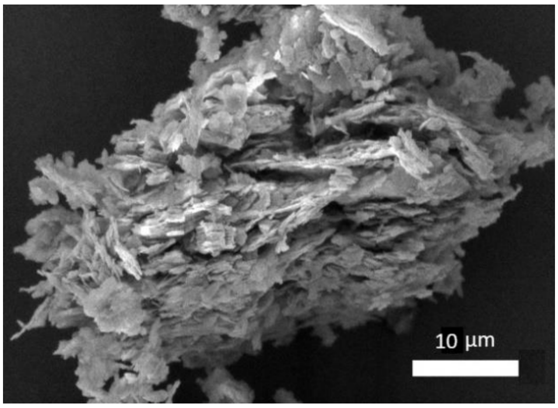


SEM images of the kaolin clay were collected to characterise the particle content. Particles / Agglomerates were found ranging from ~200nm - 20µm.

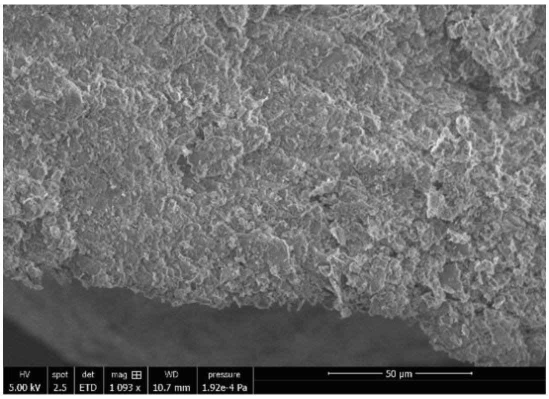


Samples of 60wt% clay were also loaded into the rheometer and pre-sheared at $20s^{-1}$. Following this a small section of the clay was cut from the sample and allowed to dry slowly in a dessicator. The samples shrink by ~10-15% during this drying process. The samples were then imaged in an SEM. The images suggest (even considering the sample shrinkage) that at these high concentrations the particles are tightly packed with perhaps some tendency to layering. One can also see some of the larger agglomerates ~ 20µm.

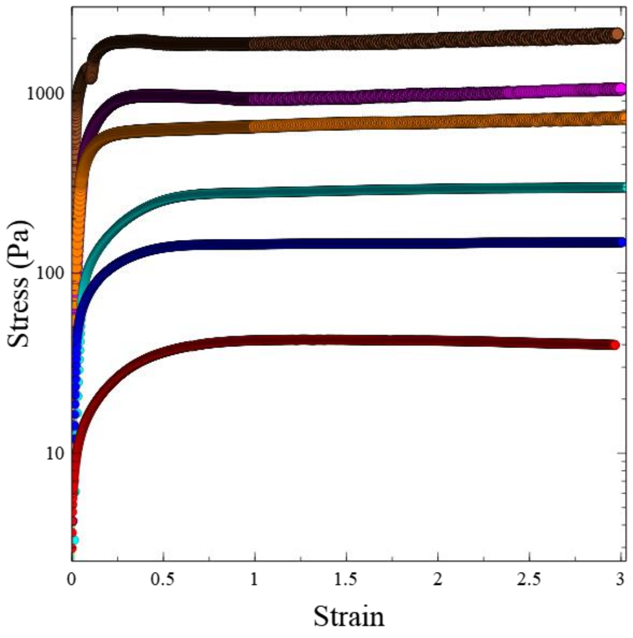


*Start-up flow measurements in simple shear using a 20mm diameter parallel plate coated with P40 sand paper at a shear-rate of 0.01s$^{-1}$. Data is shown for concentrations from bottom to top of 45,50,55,60,62.5,65 wt%.*

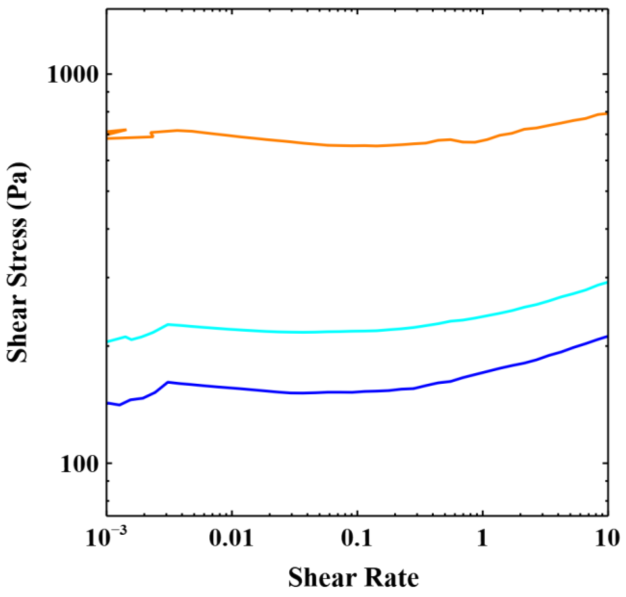


Descending shear ramp from 10 – 10$^{-3}$ s$^{-1}$ at 1 decade per minute measured using a 20mm parallel plate geometry covered with P40 sand paper. Data is shown from bottom to top for 50, 55 and 60wt% Kaolin clay.

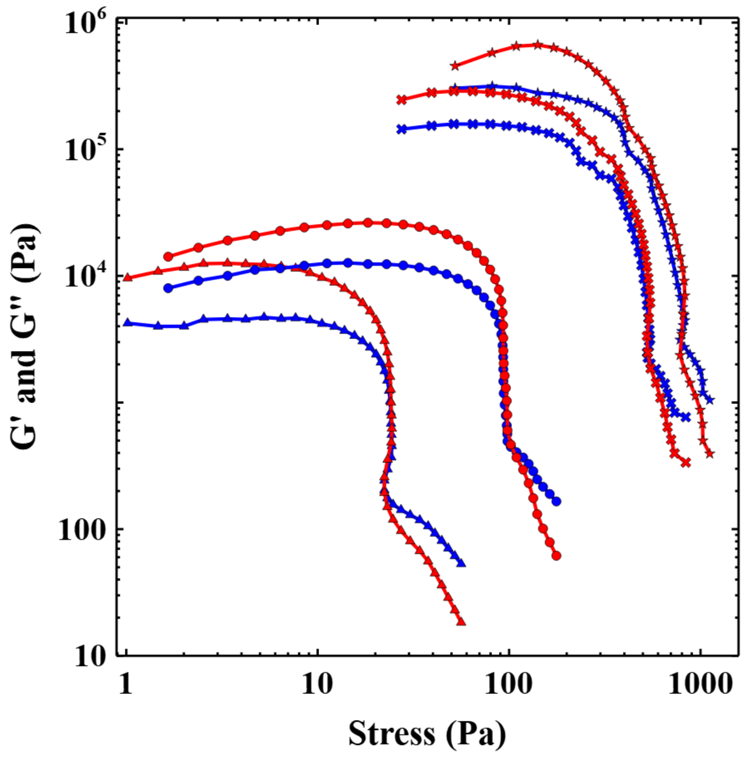


*Strain amplitude sweep at a frequency of 0.4Hz measured using a 20mm parallel plate geometry covered with P40 sandpaper. G’ (Red) and G” (Blue) measurements taken for Kaolin concentrations, left to right, 45, 50, 60 and 62.5 wt%*
